# Possible Cross-Reactivity between SARS-CoV-2 Proteins, CRM197 and Proteins in Pneumococcal Vaccines May Protect Against Symptomatic SARS-CoV-2 Disease and Death

**DOI:** 10.3390/vaccines8040559

**Published:** 2020-09-24

**Authors:** Robert Root-Bernstein

**Affiliations:** Department of Physiology, Michigan State University, East Lansing, MI 48824, USA; rootbern@msu.edu

**Keywords:** COVID-19, SARS-CoV-2, pneumococcal, Streptococcus pneumoniae, vaccine, vaccination, cross-reactivity, similarity, protection, CRM197, PspA, PsaA, PspC, BCG, poliovirus, measles–mumps–rubella, diphtheria–tetanus–pertussis, meningococcus

## Abstract

Various studies indicate that vaccination, especially with pneumococcal vaccines, protects against symptomatic cases of SARS-CoV-2 infection and death. This paper explores the possibility that pneumococcal vaccines in particular, but perhaps other vaccines as well, contain antigens that might be cross-reactive with SARS-CoV-2 antigens. Comparison of the glycosylation structures of SARS-CoV-2 with the polysaccharide structures of pneumococcal vaccines yielded no obvious similarities. However, while pneumococcal vaccines are primarily composed of capsular polysaccharides, some are conjugated to cross-reacting material CRM197, a modified diphtheria toxin, and all contain about three percent protein contaminants, including the pneumococcal surface proteins PsaA, PspA and probably PspC. All of these proteins have very high degrees of similarity, using very stringent criteria, with several SARS-CoV-2 proteins including the spike protein, membrane protein and replicase 1a. CRM197 is also present in *Haemophilus influenzae type b* (Hib) and meningitis vaccines. Equivalent similarities were found at lower rates, or were completely absent, among the proteins in diphtheria, tetanus, pertussis, measles, mumps, rubella, and poliovirus vaccines. Notably, PspA and PspC are highly antigenic and new pneumococcal vaccines based on them are currently in human clinical trials so that their effectiveness against SARS-CoV-2 disease is easily testable.

## 1. Introduction

Various studies have indicated that some vaccines may protect against symptomatic SARS-CoV-2 infection and death. A very significant inverse correlation has been found between rates of pneumococcal vaccination at both the national and local population levels and rates of SARS-CoV-2 infections and death [1]. The study found no such correlations to the tuberculosis vaccine BCG (Bacillus Calmette–Guerin), *Haemophilus influenzae type B* (Hib), diphtheria–tetanus–pertussis, measles–mumps–rubella, or poliovirus vaccinations. The results were controlled for percent of the population over 65 years of age, percent of obese individuals, percent of diabetics and the sum of these factors. Pneumococcal vaccination with PCV13 was again found to be very significantly protective in a study of 137,037 individuals for whom vaccination records were available [2] and other recent vaccinations also provided apparent protection against SARS-CoV-2 after controlling for other variables. The purpose of this paper is to provide a possible mechanism for how pneumococcal and other vaccines might protect against SARS-CoV-2.

The specific hypothesis tested here is that antigens in pneumococcal vaccines induce antibodies protective against SARS-CoV-2 by means of cross-reactivity with similar SARS-CoV-2 antigens. I have treated all other vaccines as controls. There are two types of antigens that might play such a role, one being the capsular polysaccharide antigens in current pneumococcal vaccines and the other the proteins that they contain. An extensive search for polysaccharide structures comparing SARS-CoV-2 glycosylated proteins [3] and S. pneumoniae serotypes [4] failed to identify any obvious similarities. SARS-CoV-2 glycosylations are composed mainly of various arrangements of N-acetylglucosamine, mannose, galactose and N-acetylneuraminic acid, with fucose appearing in about half of the polysaccharides [3]. While N-acetylglucosamine and some mannose derivatives appear in pneumococcal polysaccharides, N-acetylneuraminic acid does not appear in any, and only pneumococcal serogroups 4, 5, 12 and 46 contain polysaccharides composed of both mannose and fucose or N-acetylglucosamine and fucose [4]. These pneumococcal polysaccharides do not, however, appear to share any obvious structural similarities with SARS-CoV-2 polysaccharides. While the identity of polysaccharide structures is probably not required for antigenic cross-reactivity, with no obvious structural homologies, the search then shifted to possible protein similarities.

While current pneumococcal vaccines are composed primarily of capsular polysaccharides, they also contain one or both of two types of proteins. The polysaccharide component is never pure, generally containing around three percent of the cell surface proteins to which the polysaccharides are attached [5,6,7]. Proteins identified in pneumococcal vaccines include pneumococcal surface protein A (PspA) and pneumococcal surface adhesin A (PsaA) [8,9]. Because the presence of PsaA was identified only by immunological methods and PsaA cross-reacts strongly with an additional pneumococcal surface protein, PspC (also known as CbpA and SpsA) [10,11], it is likely that PspC is also present in capsular polysaccharide-based pneumococcal vaccines. Additionally, pneumococcal conjugate vaccines covalently attach the polysaccharides to a modified diphtheria toxin protein called Cross-Reactive Material 197 (CRM197), which is also present in Hib and meningitis vaccines [12].

This study investigates whether SARS-CoV-2 proteins contain regions that mimic sequences within pneumococcal surface proteins and/or CRM197 (which is also found in *Haemophilus influenzae* type B (Hib) vaccine and meningitis vaccine). Other vaccines such as measles, mumps, rubella, polio, mycobacteria and pertussis are investigated as controls.

## 2. Materials and Methods

**Figure 1:**
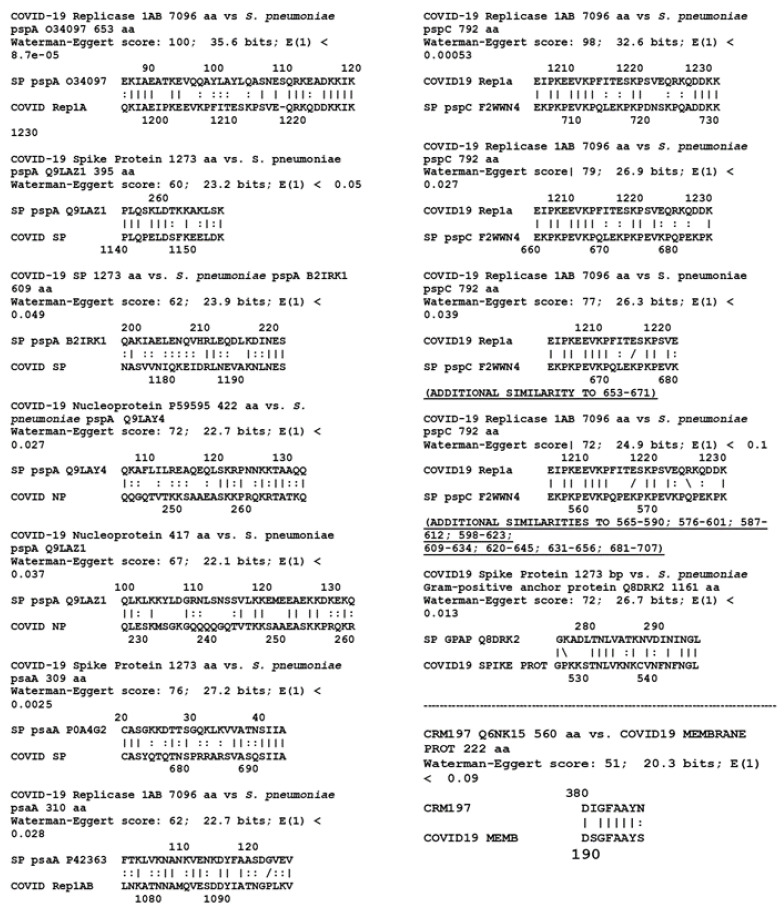
Similarities between the four known or probable pneumococcal vaccine protein contaminants PsaA, PspA, PspC, Gram-positive anchor protein and SARS-CoV-2 proteins, as well as CRM197, the modified diphtheria toxin to which pneumococcal conjugate vaccines are attached. Multiple variants for each protein were examined and the results provided here are representative of results at E = 0.1.

In order to ascertain whether PspA, PsaA, PspC and CRM197 have regions of significant similarity to SARS-CoV-2 proteins, LALIGN (at www.expasy.org) was employed to perform pair-wise protein comparisons. The parameters chosen were the 20 best alignments to show; BLOSUM80 (in order to maximize small, local similarities); E = 10; the gap penalty of −10.0 (to maximize continuous sequence similarities as are recognized by human leukocyte antigens and T cell receptors). SARS-CoV-2 sequences were retrieved from https://viralzone.expasy.org/8996 as HTML files or using the accession numbers from the UniProtKB database (UniProtKB accession numbers P0DTC1–P0DTC9). *Streptococcus pneumoniae* PspA, PsaA and PspC sequences were retrieved as accession numbers (provided in the Tables below) from the UniProtKB database. Because different streptococcal serotypes have slightly different versions of these proteins, several were randomly selected for each search and the sequences’ similarities displayed in Figure 1 are representative of several serotype results. The accession numbers for the pneumococcal vaccines, CRM197 and the control vaccine proteins are listed in Table 1.

The LALIGN results were culled by applying the criterion that any sequence similarity reported must have an E value of less than either 0.1 (Table 2) or 1.0 (Table 3), a Waterman–Eggert score of more than 50, and a region containing at least six out of ten identities. These criteria are based on a number of experimental studies involving the average length of peptide recognized by major histocompatibility (MHC) receptors and T cell receptors (TCR), which is about 10 consecutive amino acids [13,14,15], and the degree of similarity between two antigens that is likely to induce cross-reactive immune responses, which generally consists of at least five consecutive identical amino acids or six identities distributed within a 10 amino acid sequence [14,16,17,18,19,20].In essence, setting the E value to 0.1 or 1.0 determines how many matches the BLAST program will yield. The lower the E value, the less matches BLAST will yield because a lower E value limits the matches to those with rare combinations of amino acids such as methionines, tryptophans, tyrosines, cysteines, etc., rather than ones made up of sequences of very common amino acids such as glycine, alanine, valine and leucine, which appear at high rates in almost all proteins. In this case, keeping the E value low also selects for matching sequences that have a high probability of being antigenic since the immune system is more sensitive to rare amino acids than to common ones. Conversely, the lower the Waterman–Eggert score, the less amino acid matches are likely to be found in a pair of sequences. Thus, limiting the Waterman–Eggert score to more than 50 provides reasonable assurance that any sequence that appears in the BLAST search will display a high proportion of amino acid identities and similarities. Experience shows [16,17,18,19,20] that the combination of low E value and high Waterman–Eggert score tends to yield reasonably short sequences of high similarity, which is emphasized by using BLOSUM80. Despite using these boundary conditions, however, experience shows that about half of the sequences that BLAST yields are unlikely to be antigenically cross-reactive. As noted above, TCR and MHC recognize short peptide sequences averaging about 10 amino acids in length [13,14,15] and experimental evidence has shown that within such peptides, sequences of five contiguous identical amino acids or six non-contiguous amino acids are generally required for two peptides to elicit cross-reactive T cell or B cell responses [14,16,17,18,19,20]. Thus, the BLAST results were culled for sequences meeting the latter criteria. By employing the tried-and-tested set of parameters just described, previous experimentation demonstrates that the resulting matches have a high probability of being recognized as cross-reactive antigens.

As controls for the LALIGN results, all thirteen SARS-Cov-2 proteins were used to search for similarities to bacterial proteins found in diphtheria, pertussis, and tetanus vaccines (Table 1) and viral proteins incorporated into the measles, mumps, rubella and polio vaccines. The only identified proteins in Hib and meningitis vaccines are CRM197 or meningococcal outer membrane complex protein, so these were also examined for similarities to SARS-CoV-2 proteins (Table 1 and Table 2). The same criteria used above were used to screen the results for sequences having at least six identities in a span of ten amino acids.

Bacillus Calmette–Guerin (BCG) vaccine could not be searched with the other vaccines. BCG is a version of *Mycobacterium bovis* consisting of 3891 proteins. It has no integrated, searchable proteome on BLAST (www.expasy.org); instead, each protein is separately listed in the UniProt database (https://www.uniprot.org/uniprot/?query=taxonomy:410289). *M. tuberculosis* ([MYCTU_UP000001584] *Mycobacterium tuberculosis* (strain ATCC 25618/comprised 3997 sequences) was substituted for BCG since they are highly cross-reactive. Since searching nearly 4000 proteins using the LALIGN method listed above was unreasonable, the complete proteome was searched instead and BLAST was used with the parameters set similarly (BLOSUM80; E = 10; filter low complexity regions; no gaps permitted; show best 100 matches). As with the other microbial comparisons, the results were hand curated to eliminate any sequences failing to meet the six-in-ten antigenic-cross-reactivity criterion and an E value of less than 1.0 (rather than 0.1, because this value gave equivalent length and quality of matches to the LALIGN searches) and a Waterman–Eggert score of at least 50.

*Bordetella pertussis* vaccines come in two forms; one is acellular (which is the form tested above using LALIGN) but there are also whole-cell pertussis vaccines, so the same BLAST procedure used to examine *M. tuberculosis* was used to examine *Bordetella pertussis* UP000002676. Taxonomy, 257313—(strain Tohama I/ATCC BAA-589/NCTC 13251) comprised 3260 protein sequences.

As controls for the whole-bacteria BLAST searches, two human commensal bacteria, *Escherichia coli* (Escherichia coli K12 UP000000625, 4403 protein sequences) and *Clostridium leptum* ([UP000018168] *Clostridium leptum* CAG:27 proteome, 2482 protein sequences), as well as the probiotics *Lactococcus lactis* ([LACLA_UP000002196] *Lactococcus lactis* subsp. lactis (strain IL1403) 2225 protein sequences) and *Lactobacillus paracasei* ([LACP3_UP000001651] *Lactobacillus paracasei* strain ATCC 334/BCRC, 2708 protein sequences), were tested for similarities to SARS-CoV-2 proteins.

## 3. Results

**Figure 2:**
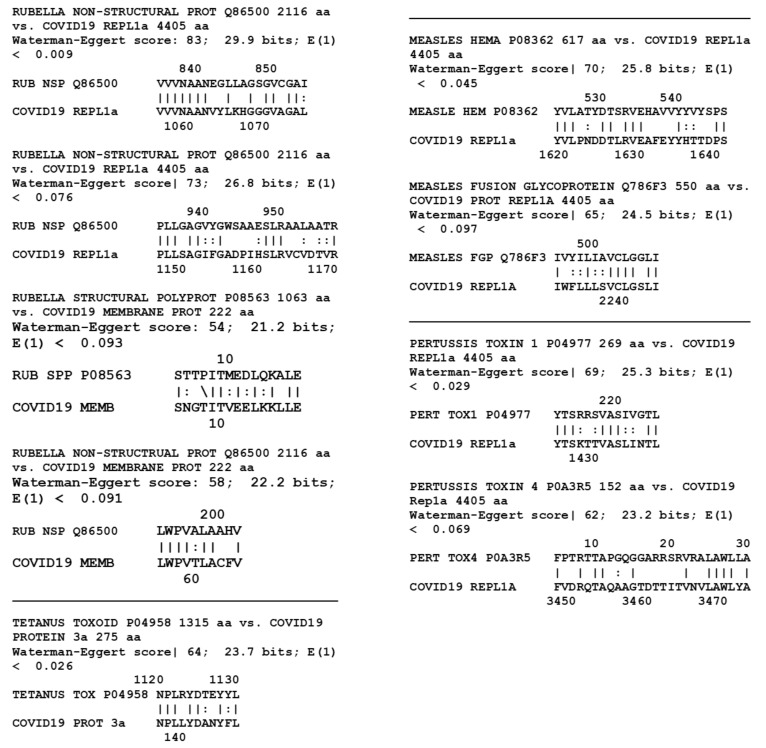
Similarities between nine SARS-CoV-2 proteins and 32 proteins from measles, mumps, rubella, polio, *Haemophilus influenzae type B* (Hib), meningitis, diphtheria, pertussis and tetanus vaccines (Table 1). A total of 288 pairwise combinations were searched. Only similarities satisfying the criteria laid out in the Methods section are shown with E = 0.1.

**Figure 3:**
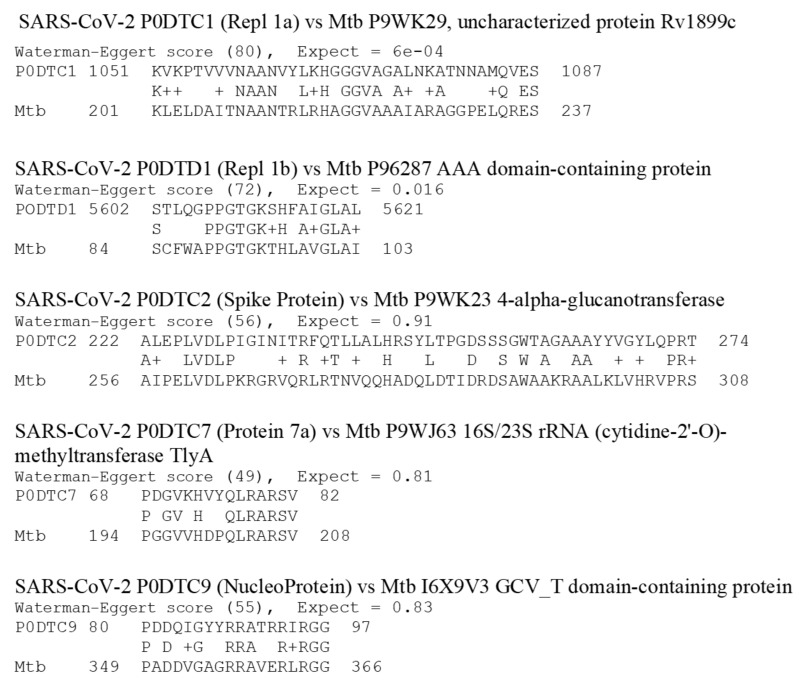
SARS-CoV-2 protein similarities with Mycobacterium tuberculosis (Mtb). Note that BCG, unlike the vaccines in Figure 1 and Figure 2 that are composed of one to seventeen proteins, is composed of 3993 proteins, so that even given the somewhat larger number of significant similarities displayed here, the probability of them being major antigens is extremely small. Note also that because of the size of the BCG proteome, BLAST (rather than LALIGN, as in Figure 1 and Figure 2), was used to find these similarities, and a cut-off value for significance of E = 1.0 rather than 0.1 was used.

**Figure 4:**
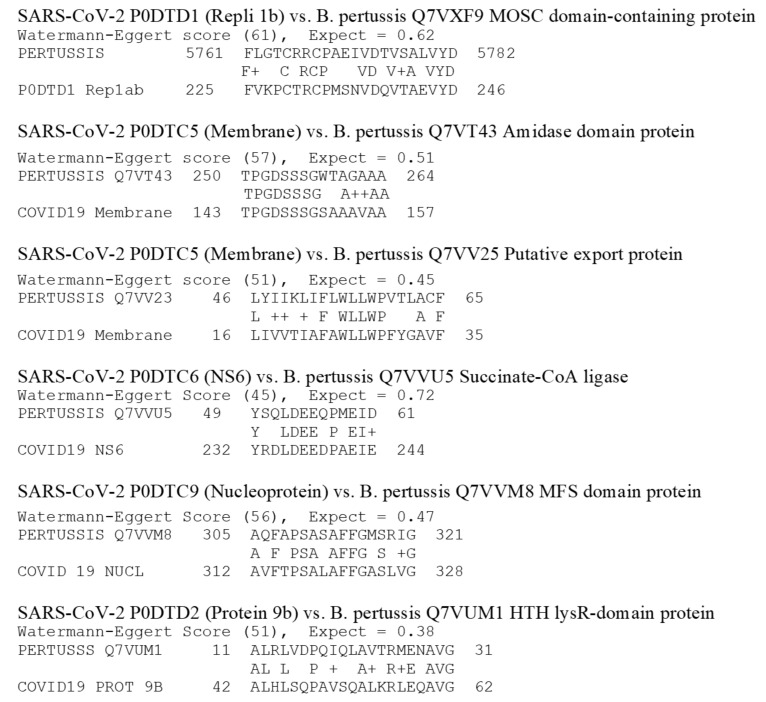
SARS-CoV-2 protein similarities with Bordetella pertussis polyprotein (UniProte accession number UP000002676). Note that whole B. pertussis is used as a vaccine. It is composed of 3260 proteins so that the probability that the matches shown are major antigens is extremely small. Note also that because of the size of the size of the B. pertussis proteome, BLAST (rather than LALIGN, as in Figure 1 and Figure 2), was used to find these similarities, and a cut-off value for significance of E = 1.0 rather than 0.1 was used, as was the case with M. tuberculosis (Figure 3) as well.

Results of the LALIGN similarity searches that satisfy the criteria of at least six identical amino acids in a sequence of ten amino acids and a Waterman–Eggert (W–E) score of 50 or greater are found in Table 2 and Table 3 and in the Figures. Results with E values of 0.1 or less are summarized in Table 2 and Figure 1, Figure 2, Figure 3 and Figure 4. Those that satisfy a W–E score of 50 or greater and an E value of 1.0 or less are summarized in Table 3 but sequences are not provided as they are too numerous.

Table 2 demonstrates that pneumococcal proteins psaA, pspA and pspC present a very large number of high-quality sequence matches with various SARS-CoV-2 proteins. All of these matches are provided in Figure 1. Twenty-one significant similarities were observed, ten of which are indicated in the figure in bold type as sequences that repeat within pairs of proteins. Note that a significant sequence similarity was also found between SARS-CoV-2 proteins and the *S. pneumoniae* Gram-positive anchor protein (Q8DRK2), which serves as an anchor site for capsular polysaccharides. It is not known at this time whether this protein is among those contaminating capsular polysaccharide preparations, but because of its association with polysaccharide anchoring, it is likely to be such a contaminant of the polysaccharide material used in pneumococcal vaccines. Each of the four streptococcal proteins was tested against each of the SARS-CoV-2 proteins, yielding 52 pairwise tests. Six of these combinations yielded one or more matches that satisfied all similarity criteria employed here. An additional 30 matches between these pneumococcal proteins and SARS-CoV-2 proteins were found when E was relaxed to 1.0 (Table 3) for a total, including the CRM197 matches, of 61.

One significant match at E = 0.1 was also found between CRM197 and the membrane protein (P0DTC5) of SARS-CoV-2 (Table 2 and Figure 1) with an additional nine matches at E = 1.0 (Table 3). However, there were no significant similarities at E = 0.1 between the meningococcal outer membrane protein complex and any SARS-CoV-2 protein (Table 2), and only five when E was relaxed to 1.0 (Table 3).

Figure 2 displays the results for the pairwise tests of the thirteen SARS-CoV-2 proteins with the additional bacterial and viral proteins listed in Table 1 that are present in measles, mumps, rubella, polio, diphtheria, pertussis, and tetanus vaccines, for a total of 32 microbial proteins. Of these, six yielded one or more significant similarities for a total of nine matches out of 416 possible pairwise combinations (Table 2). When the E value was relaxed to 1.0 (Table 3), an additional 81 matches were found, most notably between rubella vaccine proteins and SARS-CoV-2 proteins.

Results from the BLAST searches on whole bacteria are presented in Table 4. The 3997 M. tuberculosis proteins yielded five significant similarities at an E value of 1.0 or less when compared with the 13 SARS-CoV-2 proteins (51,961 combinations) (Figure 3). These matches are of roughly equivalent quality to those of the LALIGN searches conducted on the other vaccine proteins described above. The sequences are listed in Figure 3. Raising the E value to 10 and lowering the Waterman–Eggert (W–E) score to 40 increased the total number of matches (still including at least six identities in a stretch of 10 amino acids) to 36. These matches appear to be equivalent in quality to those found for E = 1.0 for the LALIGN searches. Similarly, the whole pertussis proteome (3260 proteins) yielded only six matches at E = 0.1 and the W–E score at 50 (Table 2 and Figure 4), which increased to 55 when the W–E score was lowered to 40 and E was raised to 1.0 (Table 3). However, these results do not differ significantly from those obtained from commensal and probiotic control bacteria (Table 4): the average number of matches per protein for the tuberculosis and pertussis bacteria at E = 1.0 was 0.0015 and at E = 10.0, 0.013, whereas the average number of matches per protein for the control bacteria at E = 1.0 was 0.0015, and at E = 10.0, 0.014. These results suggest that the rate of matches between *M. tuberculosis* and SARS-CoV-2 is what can be expected as the result of randomness rather than any of the tested bacteria expressing particular protein sequences of relevance to the current study.

Large differences in the number of matches was found between pneumococcal proteins and those from other protein antigen vaccines for the LALIGN E = 0.1 group (Table 2, Figure 1 and Figure 2). All four of the pneumococcal proteins and the CRM197 protein had significant similarities (i.e., meeting the similarity criteria laid out in the Methods) to at least one of the thirteen SARS-CoV-2 proteins. Altogether, seven of the 65 possible permutations of pneumococcal protein pairs yielded significant similarities, or 10.8 percent. In contrast, only eight of the 35 viral and bacterial vaccine proteins other than whole-cell pertussis and *M. tuberculosis* had significant matches to any of the nine SARS-CoV-2 proteins (1.8% of the 455 pairwise comparisons). The four pneumococcal proteins yielded 21 significant matches with SARS-CoV-2 proteins, for an average of 5.25 per pneumococcal protein, while the 35 other vaccine proteins yielded only nine significant matches, for an average of 0.26 per protein. In other words, at the E = 0.1 criterion, the probability of a match leading to cross-reactivity is over 20 times more likely for pneumococcal proteins than for those from other vaccines.

The E = 1.0 data (Table 3) yielded similar results. The pneumococcal proteins exhibited a total of 61 matches (including CRM197) with SARS-CoV-2 proteins for an average of 12.2 matches per protein. The rest of the vaccines (other than whole cell pertussis and BCG) exhibited 90 total matches spread out over 35 proteins for an average of 2.5 matches per protein. The 61 pneumococcal matches were found among 23 of the 65 permutations with SARS-CoV-2 proteins, or 35.2 percent. In contrast, the 90 other vaccine matches were spread out over 53 of the 455 pairwise permutations, representing 11.6 percent of the possibilities. In other words, using the E = 1.0 criterion as a cutoff, it is three times more likely that pneumococcal proteins will result in a cross-reactive match than for other proteins. In this instance, rubella antigens account for more than thirty percent of the non-pneumococcal matches, making rubella the next best candidate for protecting against SARS-CoV-2 infection.

The whole cell vaccines were treated separately from the limited antigen vaccines because BLAST was used rather than LALIGN to perform the searches and because the average number of individual vaccine protein matches to SARS-CoV-2 was very different: for the whole cell bacteria, it was 0.0145 with an SD of 0.0055, whereas the individual vaccine proteins (using the E =1.0 data in Table 3) is about 5.4 with a standard deviation of 2.6. For *M. tuberculosis*, for example, the best rate of matches was 40 out of 51,961 combinations [E = 10], or 0.08 percent, with an average of one match per 100 M. tuberculosis proteins. At worst, using E =1.0, there were only 5 matches out of 51,961 combinations or 0.01 percent, with one match per every 800 M. tuberculosis proteins. The pertussis results were very similar. On a per-protein basis, these two bacteria resulted in rates of matches that were two orders of magnitude lower than the other proteins tested (Table 2 and Table 3). Thus, the percent of whole-bacteria matches (Table 4) is clearly very much lower than the percent of matches for the limited-antigen vaccines listed in Table 2 and Table 3. The paucity of matches on a per-protein basis resulting from the tuberculosis and pertussis bacteria comparisons is itself noteworthy, strongly suggesting that the quality of matches reported in Figure 1 and Figure 2 for the other vaccines are intrinsically extraordinary, and the pneumococcal (both E = 0.1 and E = 1.0) and rubella (E = 1.0) results particularly so.

## 4. Discussion

The results of this study indicate that while pneumococcal vaccines are primarily composed of polysaccharides, there are no obvious structural homologies between these polysaccharides and SARS-CoV-2 glycosylations. The absence of such homologies does not rule out antigenic cross-reactivity between these polysaccharides, but makes their identification difficult using anything other than direct tests of whether SARS-CoV-2 antibodies recognize pneumococcal polysaccharides or whether pneumococcal antibodies recognize SARS-CoV-2. Such tests might be worth conducting if only as controls for studies of possible cross-reactivity between proteins found in pneumococcal vaccines and SARS-CoV-2 proteins.

CRM197, which is used to conjugate pneumococcal polysaccharides in conjugate vaccines such as the Prevnar series, and pneumococcal proteins known to contaminate the vaccines significantly, both mimic SARS-CoV-2 proteins (Figure 1), satisfying rigid similarity and antigenicity constraints, though there are many more high-quality matches between the pneumococcal proteins than with CRM197. The results point specifically to potential cross-reactivity between SARS-CoV-2 proteins and the pneumococcal proteins PspA and PsaA, which are known to contaminate polysaccharide-based pneumococcal vaccines [5,6,7] as well as PspC, which it is reasonable to assume is another such contaminant since it derives from the same outer membrane protein complex and is highly cross-reactive with the antibodies against PspA used to demonstrate the presence of PspA in vaccines [10,11]. Such cross-reactivity would be consistent with epidemiological studies suggesting a protective effect of pneumococcal vaccination against SARS-CoV-2 [1,2]. Since the CRM197 protein is used to conjugate some *Haemophilus* and meningitis vaccines, these vaccines may also provide some cross-reactive protection against SARS-Cov-2 proteins (Figure 1), a result that is consistent with the findings of Pawlowski, et al. [2]. Further clinical and experimental tests of whether these vaccines elicit antibodies that are cross-reactive with SARS-CoV-2 proteins are clearly needed.

It is important to emphasize that the fact that a microbe expresses an antigen that is sequentially similar to a SARS-CoV-2 protein is not sufficient to guarantee that the two will elicit cross-reactive immunity or, for that matter, any immune response whatsoever, since that sequence may not be processed as an antigen by macrophages or presented to T cells. The full range of determinants of antigenicity are as yet unknown. Among the key factors seem to be the concentration of the antigen, how dissimilar it is from its host, where the antigen is expressed within a protein (e.g., whether it is freely accessible in a random loop or protected within a pleated beta sheet), how the antigen is presented to the immune system (e.g., by ingestion, inoculation, infection), and the inflammatory context in which the antigen is processed (e.g., in the presence of an adjuvant or bystander infection) [21]. In this context, it is notable that the rate of SARS-CoV-2 matches to pneumococcal vaccine antigens is 14%, so that there is a reasonable chance of the immune system encountering a cross-reactive antigen, as is the case for CRM197-conjugated vaccines and rubella and measles, whereas the rates of matches to mycobacterial and pertussis antigens are very low (less than 0.1%). However, the data presented here do not rule out the possibility of cross-reactivity to pertussis and/or mycobacterial proteins, though it is striking that the rate of SARS-CoV-2 matches to pertussis and mycobacterial proteins is not significantly different than to the commensal and probiotic control bacteria *L. lactis, L. paracasei, C. leptum* and *E. coli* (Table 4). On the one hand, these data could be interpreted to mean that mycobacteria and pertussis are as unlikely as the commensal and probiotic bacteria to protect against SARS-CoV-2. On the other hand, if mycobacteria or pertussis bacteria are sufficient to induce protection against SARS-CoV-2, one might argue that these commensal and probiotic bacteria could do so as well; since everyone encounters them, protection against SARS-CoV-2 should then be universal. However, several factors undermine this latter conclusion. One is that the mode of presentation of vaccines to the host immune system is very different than that of commensal and probiotic bacterial antigens. Vaccines either actively infect the host (e.g., polio or influenza), or are (more often) inoculated at very high concentrations; in both cases, the resulting tissue damage initiates an immune response. Probiotic and commensal microbes, in contrast, are retained (in healthy people) in the gut and do not cause tissue damage or initiate an active immune response [22]. Additionally, the immune system often develops tolerance for commensal and probiotic organisms and such organisms express large numbers of antigens that mimic host antigens, including T cell receptors and human leukocyte antigens, thereby camouflaging themselves from immune surveillance [23,24,25,26,27]. Thus, while any given commensal or probiotic microbe has some small probability of expressing antigens that could potentially protect against SARS-CoV-2 infection, their general inability to elicit active immunity militates against this being a likely scenario.

The concentration of antigen presented to the immune system is also a determinant of whether an active immune response results, so that microbes expressing very large numbers of antigens in very small quantities are unlikely to elicit a strong immune response to most of them. The concentration of protein contaminants in pneumococcal vaccines is clearly sufficient to induce immunity. CRM197 is present in equal amounts to the capsular polysaccharides in the vaccines and is present because it is known to be highly antigenic. In Prevnar-13, for example, there are 30.4 µg of capsular polysaccharides and 34.0 µg of CRM197 for a total of 64.4 micrograms of antigen per dose [28]. Protein contaminants may make up an additional 3%, or 1.92 µg, of antigenic material according to WHO guidelines and confirmed by laboratory analysis [5,6,7]. This 1.92 µg of protein is virtually identical to the 2.2 µg of each of twelve of the capsular polysaccharides present (plus 4.4 µg of serotype 6) or the 2.3 micrograms of CRM197 conjugated to each polysaccharide type [28] and is therefore sufficient to induce an immune response, especially since PspA and PspC are strongly antigenic and cross-reactive. Pneumovax-23, in contrast, has 25 µg of each capsular polysaccharide, adding up to a total of 575 µg of antigen [29]. The three percent protein contamination allowed by the WHO [5,6,7] could result in 17.25 µg of total PsaA, PspA and PspC per dose, which is certainly sufficient to induce immunity. For comparison, each 0.5-mL dose of Adacel^®^, a diphtheria–tetanus–pertussis vaccine (Sanofi Pasteur), contains only 2.5 µg detoxified pertussis toxoid, 5 µg FHA, 3 µg pertactin and 5 µg FIM acellular pertussis antigens [30].

In addition to being present in concentrations that could induce protective immunity, the pneumococcal-SARS-CoV-2 similarities reported here satisfy multiple criteria involving sequence identities and search parameters for predicting potential antigenic cross-reactivity, so it is possible that pneumococcal vaccination can protect individuals against SARS-CoV-2 disease. Evidence of protection against SARS-CoV-2 by T cells reactive to unidentified, cross-reactive microbes has been reported by several groups [31,32,33]. The studies report that 40 to 60% of people unexposed to SARS-CoV-2 had SARS-CoV-2-reactive CD4+ T cells. This cross-reactivity is proposed to result from prior exposure to coronaviruses that cause colds but this hypothesis has not yet been tested [33]. Moreover, the studies also report that this cross-reactive immunity is greatest in young people and least in older people, which is not consistent with cold virus exposures, nor is the fact that over 90% of people have T-cells that are reactive to cold viruses but few seem to be immune to SARS-CoV-2 [33]. Such waning immunity is, however, consistent with waning childhood vaccination immunity and particularly for vaccinations such as pneumococci that are not universal. In light of the data presented here, it is therefore possible that at least some proportion of individuals with cross-reactive immunity developed it through exposure to pneumococcal vaccinations. Such cross-reactivity would also explain the epidemiological observation that pneumococcal vaccination rates correlate inversely with rates of serious SARS-CoV-2 disease and death, but that vaccination rates with other commonly used vaccines (DTP, MMR, polio, meningitis, and BCG), do not [1].

One might ask whether the immunity conferred by pneumococcal (and perhaps other) vaccines is sufficient to prevent SARS-CoV-2 infection completely. The current study is incapable of addressing that question meaningfully but the fact that the vast majority of similarities between pneumococcal proteins and SARS-CoV-2 involve the replicase (Table 2 and Table 3) suggests that any protection would be reactive rather than preventative. The reason for this is that the replicase is not expressed until cells are infected so that pneumococcal-related immunity would mainly come into play only at that point. This factor might explain why many people seem to become infected with SARS-CoV-2 and remain infectious without themselves displaying symptoms of COVID-19 [34,35,36,37,38]. Indeed, increasing evidence indicates that the primary protection against SARS-CoV-2 is T-cell-mediated rather than antibody-mediated [31,32,33], suggesting that control of the infection is at the level of cellular infection rather than against free virus. Nonetheless, it is notable that the next most prevalent set of SARS-CoV-2–pneumococcal protein similarities after the replicase involve the viral spike protein (Table 2 and Table 3), which is a major target for antibodies and which might, therefore, mediate SARS-CoV-2 infectivity.

The observation that viral and bacterial proteins exhibit antigens similar enough to be cross-reactive may be surprising but it is not novel. Härkönen, et al. [39] found that rabbit antibodies to HSP65 of *Mycobacterium bovis* (from which BCG is derived) recognized capsid protein VP1 of coxsackievirus A9, VP1, and/or VP2 of coxsackievirus B4. Misko, et al. [40] demonstrated that Epstein–Barr virus mimicked a *Staphylococcus aureus* replication initiation protein and induced antibodies cross-reactive with it. Trama, et al., [41] and Williams, et al. [42] have documented antibodies against the gp41 protein of human immunodeficiency virus that cross-react with commensal bacteria in the human gut. Ross, et al. [43] reported that sera from chickens inoculated with infectious bursal disease viruses or infectious bursal disease vaccines cross-reacted with *Mycoplasma gallisepticum* and *Mycoplasma synoviae*. In addition, Bordenave [44] found that antibodies against *Salmonella abortusequi* also recognized tobacco mosaic virus. In short, while the phenomenon may be rare—and, indeed, the data reported here suggests that such similarities may occur at a rate as high as 1/70 pairwise protein combinations or as low as 1/1000—bacterial antigens are known to occasionally induce antibodies that cross-react with viral antigens or vice versa. This observation is consistent with the fact that every possible sequence of five amino acids has been shown to appear randomly in the microbial proteome [45,46]. Completely unrelated microbes should, therefore, have a small, but finite, probability of expressing identical antigens capable of inducing cross-reactive immune responses. The question becomes one of whether these antigens are ever encountered by the host and presented to the immune system in a way that initiates cross-reactive immunity.

The almost completely negative results reported here for antigenic mimicry between SARS-CoV-2 proteins and proteins from measles, mumps, diphtheria, pertussis and tetanus at E = 0.1 (Table 1), and the relatively low rate of similarities with poliovirus at E = 1.0 (Table 2), are consistent with the lack of association between these vaccines and SARS-CoV-2 rates of disease or death [1], although Pawlowski, et al. [2] found some protective effect from polio vaccination and the measles–mumps–rubella (MMR) combination vaccine. The current study would suggest that the rubella component of MMR is the major protective agent, though measles also exhibits some high-quality antigenic similarities to SARS-CoV-2. Indeed, Franklin, et al., [47] also report significant similarities between both rubella and measles proteins and SARS-CoV-2, and their key results were independently reproduced here in Figure 2. Additionally, Gold [48] has also proposed that the measles–mumps–rubella vaccine may confer protection against SARS-CoV-2. However, there are significantly fewer similarities between measles and rubella proteins and those of SARS-CoV-2 proteins (and none with mumps proteins) than there are with pneumococcal proteins, making pneumococci a much higher probability source of protection. Moreover, epidemiological evidence does not support measles containing vaccines (which often include rubella) as protective against SARS-CoV-2, though using measles-containing vaccines as Root-Bernstein [1] did, may hide important rubella-related protection since not all measles-containing vaccines include rubella and rubella vaccination can be performed independently from measles vaccination. The suggestion that the polio vaccine be tested as a SARS-CoV-2 vaccine [49] is likewise not well-supported by either the data presented here, which found only one significant similarity between polio proteins and SARS-CoV-2 proteins at E = 0.1 and five at E = 1.0 (Table 1 and Table 2 and Figure 2), or by epidemiological data [1], though, once again, Pawlowski, et al. [2] found some protective effect in children.

The data presented here must be interpreted both probabilistically—which is to say as a guide to whether any particular vaccine has a greater or lesser probability of providing antigens that are both cross-reactive and protective against SARS-CoV-2 infection or complications—and antigenically, which is a measure of how strong an immune response a sequence actually elicits. Using both criteria, pneumococcal vaccine antigens are the most probable candidates for providing such protection since there are many matches and the pneumococcal proteins are known to be highly antigenic. The rubella antigens are the next most likely for the same reasons. However, we cannot know for certain until the appropriate immunological cross-reactivity studies are conducted to determine both whether antibodies against the vaccine antigens recognize SARS-CoV-2 antigens and protect against infection, and whether SARS-CoV-2 antibodies recognize the potentially cross-reactive antigens identified in Figure 1, Figure 2, Figure 3 and Figure 4.

The criteria just described apply equally to considerations of whether there is cross-reactivity to the BCG vaccine. Tuberculosis (BCG) vaccination has also been proposed to protect against SARS-CoV-2 [50]. While BCG vaccination was purported to be associated with SARS-CoV-2 protection in several epidemiological studies (reviewed in [51]), that result was not replicated in others (e.g., [1,2,52]) and serious concerns about methodologies have called into question the association [51,53]. The current study leads to the conclusion that BCG protection against SARS-CoV-2 is unlikely. While between 5 (E = 0.1) and 40 (E = 1.0) similarities were found between *M. tuberculosis* proteins and SARS-CoV-2 proteins, this number is insignificant in relation to the number of proteins expressed by *M. tuberculosis* and BCG (approximately 4000). This paucity of significant *M. tuberculosis similarities* (0.04%) as compared with the high incidence of pneumococcal similarities (11.6–14%) makes it probable that pneumococcal proteins will induce cross-reactive antibodies and extremely unlikely that any of the *M. tuberculosis* antigens will do so. Indeed, none of the *M. tuberculosis* proteins identified in Figure 3 are among the known dominant antigens expressed by either *M. tuberculosis* infection or BCG vaccination [54,55,56,57,58].

The question of whether pertussis antigens may protect against SARS-CoV-2 is more complicated than that for BCG. There appear to be no epidemiological studies associating pertussis vaccination with protection against SARS-CoV-2 infection or death and the one study that has looked for such an association found none [1]. However, while acellular pertussis vaccines have a very small number of sequences that are potentially cross-reactive with SARS-CoV-2 proteins, the whole cell vaccine, which is still available in some countries, has many matches specifically to the SARS-CoV-2 spike protein, which is a major target of neutralizing antibodies (Table 4). The difficulty is that with 3260 proteins in the whole cell vaccine, the probability that any of these potentially cross-reactive sequences are actually processed as major antigens inducing significant antibody responses is small, particularly compared to pneumococcal and rubella vaccines (Table 1 and Table 2). However, some of these proteins have been incorporated into the acellular pertussis vaccines and are known to be highly antigenic. Thus, the total number of matches is probably a less useful predictor of antigenic cross-reactivity than whether the potentially cross-reactive proteins are known to be highly antigenic, as is the case with the pneumococcal and rubella proteins. Again, theory can be a guide here, but experiment will provide the final answers.

Finally, it must be mentioned that the correlations between pneumococcal vaccination (and perhaps other vaccinations) and decreased risk of SARS-CoV-2 cases and deaths may be due not to cross-reactivity between pneumococcal (or other vaccine) antigens and SARS-CoV-2 antigens but rather to protection against super-infection of SARS-CoV-2 by pneumococci and other bacteria. While it is common to attribute all of the symptoms of COVID-19 to SARS-CoV-2 infection, a rapidly expanding literature is demonstrating that, as with influenza [59,60], serious COVID-19 cases are characterized by bacterial super-infections of which pneumococci, *Haemophilus influenzae* and Mycoplasmas are the most common [61,62,63,64,65,66,67,68,69]. For example, a recent study from China found that 60% of COVID-19 patients had streptococcal infections, about 55% *Klebsiella pneumoniae* infections and 40% had Hib [70]. Indeed, severe COVID-19 cases are characterized by elevated procalcitonin levels [68,69] and by eosinopenia [66,67], both of which are diagnostic for disseminated bacterial infections [71,72]. If this is the case, then pneumococcal and Hib vaccination may not prevent SARS-CoV-2 infection but should decrease the probability of developing the complications associated with severe COVID-19 disease.

## 5. Conclusions

To conclude, there are many reasons to investigate whether pneumococcal, Hib, meningitis and rubella vaccination may protect against SARS-CoV-2 infection or complications. Epidemiologically, a strong inverse association of pneumococcal vaccinations with rates of SARS-CoV-2 rates of disease and death has been documented by two studies [1,2]. The epidemiological association makes sense in terms of the particular proteins found in pneumococcal vaccines that are identified in this study as being potentially protective. These are CRM197, PspA, PsaA and PspC, all proteins known to be highly antigenic [73]. Since CRM197 is also found in Hib vaccines, which have also been associated with protection against SARS-CoV-2 [2], its cross-reactivity with SARS-CoV-2 proteins should be investigated. The other pneumococcal proteins (PspA, psaA and PspC) are under active investigation as more effective and broadly protective pneumococcal vaccine components to replace the polysaccharide-based vaccines [74,75,76,77]. Some of these vaccine candidates are already in human trials [77,78]. Thus, it should be possible rapidly and readily to determine whether such pneumococcal protein-based vaccines can be effective mitigators of SARS-CoV-2 disease and these vaccines may provide needed protection until a SARS-CoV-2 vaccine is produced in sufficient quantities to be effective worldwide. Finally, rubella vaccination should also be investigated further since rubella proteins have the second highest rate of similarities to SARS-CoV-2 proteins in this study and rubella vaccination has been reported to have some protective efficacy against SARS-CoV-2 [2].

Because pneumococcal vaccination has the highest degree of protection in both studies that have compared it with other vaccines [1,2], it seems logical to focus current efforts on this type of vaccination. Regardless of the efficacy of such pneumococcal vaccines in protecting against serious SARS-CoV-2 infection, increased use of pneumococcal vaccination should be urged because the world will be facing dual epidemic/pandemics this coming fall and winter and perhaps for many years hereafter, involving concurrent influenza and SARS-CoV-2. Increasing pneumococcal and Hib (which also contains CRM197) vaccination coverage has been demonstrated to be one of the most effective means to lower the incidence of pneumonias and intensive care unit cases following influenza infections [79,80]. At a minimum, decreasing the rates of invasive pneumococcal and *Haemophilus influenzae* superinfections following influenza infections will free up badly needed resources, personnel and intensive care units for treating SARS-CoV-2 patients. Several nations have already adopted, or are considering, policies to increase pneumococcal vaccination coverage for just this reason [81,82,83,84]. If the current research is accurate, Hib should be added to this list and nations adopting these policies may also benefit in having fewer serious SARS-CoV-2 cases because of protection from cross-reactive antigens. This is a no-lose and possibly a win-win situation.

## Figures and Tables

**Table 1:** UniProtKB accession numbers for viral and bacterial proteins used in this study.

MICROBE	UniProt Identification	List of Proteins
STREPTOCOCCUS PNEUMONIAE	O34097	pspA, Pneumococcal Surface Protein A
	Q9LAZ1	
	B2IRK1	
	Q9LAY4	
	P0A4G2	psaA, Pneumococcal surface protein, Manganese ABC transporter substrate protein
	P0A4G3	
	P42363	
	Q04JB8	
	Q9KK40	pspC, Pneumococcal Surface protein PspC
	Q9FDQ1	
	Q9KK37	
	Q9KK24	
	Q8DRK2	Pneumococcal Gram-positive anchor protein
MUMPS	P11235|	HN_MUMPM (HN)RecName: Full = Hemagglutinin-neuraminidase
	P30929	L_MUMPM (L)RecName: Full = RNA-directed RNA polymerase L
	P09458	FUS_MUMPR (F)RecName: Full = Fusion glycoprotein F0
	P30928	V_MUMPM (P/V)RecName: Full = Non-structural protein V
	P22112	SH_MUMPM (SH)RecName: Full = Small hydrophobic protein
MEASLES	P08362	HEMA_MEASE (H)RecName: Full = Hemagglutinin glycoprotein
	Q89933	NCAP_MEASF (N)RecName: Full = Nucleoprotein
	P12576	L_MEASE (L)RecName: Full = RNA-directed RNA polymerase L
	Q786F3	FUS_MEASC (F)RecName: Full = Fusion glycoprotein F0
	P0C774	V_MEASC (P/V)RecName: Full = Non-structural protein V
RUBELLA	P08563	POLS_RUBVM RecName: Full = Structural polyprotein (contains spike protein E1, spike protein E2, capsid protein) 1063 aa
	Q86500	POLN_RUBVM RecName: Full = Non-structural polyprotein p200 (contains p90, p150 and p200 proteins) 2116 aa
POLIO	P03301	P03301|POLG_POL1S RecName: Full = Genome polyprotein; 2209 aa CONTAINS: P3; Protein 3AB; P1; Capsid protein VP0; Capsid protein VP4; Capsid protein VP2; Capsid protein VP3
PERTUSSIS	P04977	TOX1_BORPE (ptxA)RecName: Full = Pertussis toxin subunit 1
	P04978	TOX2_BORPE (ptxB)RecName: Full = Pertussis toxin subunit 2
	P04979	TOX3_BORPE (ptxC)RecName: Full = Pertussis toxin subunit 3
	P0A3R5	TOX4_BORPE (ptxD)RecName: Full = Pertussis toxin subunit 4
	P04981	TOX5_BORPE (ptxE)RecName: Full = Pertussis toxin subunit 5
	P35077	FHAC_BORPE (fhaC)RecName: Full = Filamentous hemagglutinin transporter protein FhaC
	P14283	PERT_BORPE (prn)RecName: Full = Pertactin autotransporter
	P05788	FM2_BORPE (fim2)RecName: Full = Serotype 2 fimbrial subunit
	P17835	FM3_BORPE (fim3)RecName: Full = Serotype 3 fimbrial subunit
TETANUS	P04958	TETX_CLOTE (tetX)RecName: Full = Tetanus toxin
DIPHTHERIA	Q5PY51	Q5PY51_CORDP SubName: Full = Diphtheria toxin
	Q6NK15	Q6NK15_CORDI (tox)SubName: Full = Diphtheria toxin
MENINGOCOCCUS	0DH58	OMPA_NEIMB (porA)RecName: Full = Major outer membrane protein
SARS-CoV-2	P0DTC1	P0DTC1 Replicase polyprotein 1a (pp1a)
	P0DTC2	P0DTC2 Spike glycoprotein (S)
	P0DTC3	P0DTC3 Protein 3a (NS3a)
	P0DTC4	P0DTC4 Envelope small membrane protein (E)
	P0DTC5	P0DTC5 Membrane protein (M)
	P0DTC6	P0DTC6 Non-structural protein 6 (NS6)
	P0DTC7	P0DTC7 Protein 7a (NS7a)
	P0DTC8	P0DTC8 Non-structural protein 8 (NS8)
	P0DTC9	P0DTC9 Nucleoprotein (N)
	P0DTD1	P0DTD1 Replicase polyprotein 1ab (pp1ab)
	P0DTD2	P0DTD2 Protein 9b (NS9B)
	P0DTD3	P0DTD3 Uncharacterized protein 14 (NS14)
	P0DTD8	P0DTD8 Protein 7b (NS7b)
Mycobacterium tuberculosis	MYCTU_UP000001584	M. tuberculosis (strain ATCC 25618/3997 protein sequences; 1,332,562 total letters
Bordetella pertussis	BORPE_UP000002676	B. pertussis strain Tohama I/ATCC BAA-589/NCTC 13251; 3260 proteins sequences
Escherichia coli K12	ECOLI_UP000000625	Escherichia coli K12, 4403 protein sequences
Clostridium leptum	9CLOT_UP000018168	Clostridium leptum CAG:27 proteome; 2482 protein sequences
Lactobacillus paracasei	LACP3_ UP000001651	Lactobacillus paracasei strain ATCC 334/BCRC; 2708 protein sequences
Lactococcus lactis	LACLA_UP000002196	Lactococcus lactis subsp. lactis (strain IL1403); 2225 protein sequences

**Table 2:** Summary of LALIGN searches set to E = 0.1 comparing SARS-CoV-2 proteins (left-hand column) with vaccine proteins (see Table 1 for list of individual proteins). PNEUM = pneumococcal; CRM197 = Cross-Reactive Material 197; Acell PERT = acellular pertussis vaccine; DIPH = diphtheria vaccine; TET = tetanus vaccine; Whole PERT = whole cell pertussis vaccine; BCG = Bacillus Calmette–Guerin, here represented by *M. tuberculosis*. Avg/Pro = average number of matches per protein.

LALIGN E = 0.1	PNEUM	CRM 197	RUB-ELLA	MEAS-LES	MUMPS	Acell PERT	DIPH	TET	POLIO	Men-ingitis
P0DTC1 Repl 1a	15	0	2	2	0	2	0	0	0	0
P0DTC2 Spike Prot	4	0	0	0	0	0	0	0	0	0
P0DTC3 Prot 3a	0	0	0	0	0	0	0	1	0	0
P0DTC4 Env Prot	0	0	0	0	0	0	0	0	0	0
P0DTC5 Memb Prot	0	1	2	0	0	0	0	0	0	0
P0DTC6 NS6 Prot	0	0	0	0	0	0	0	0	0	0
PODTC7 Prot 7a	0	0	0	0	0	0	0	0	0	0
P0DTC8 NS8 Prot	0	0	0	0	0	0	0	0	0	0
P0DTC9 Nucleoprot	2	0	0	0	0	0	0	0	0	0
P0DTD1 Repl 1ab	0	0	0	0	0	0	0	0	0	0
P0DTD2 NS9b Prot	0	0	0	0	0	0	0	0	0	0
P0DTD3 NS Prot 14	0	0	0	0	0	0	0	0	0	0
P0DTD8 Prot 7b	0	0	0	0	0	0	0	0	0	0
Total Matches	21	1	4	2	0	2	0	1	0	0
# Proteins	4	1	6	5	5	9	1	1	7	1
Avg/Prot	5.2	1.0	0.7	0.4	0	0.2	0	1.0	0	0

**Table 3:** Summary of LALIGN searches set to E = 1.0 comparing SARS-CoV-2 proteins (left-hand column) with vaccine proteins (see Table 1 for list of individual proteins). Note that the BLAST searches on Whole PERT and BCG were set to E = 10 because of the much larger size of the entire genome as compared with the average of 17 proteins searched for in the other vaccines. PNEUM = pneumococcal; CRM197 = Cross-Reactive Material 197; Acell PERT = acellular pertussis vaccine; DIPH = diphtheria vaccine; TET = tetanus vaccine; Whole PERT = whole cell pertussis vaccine; BCG = Bacillus Calmette–Guerin, here represented by M. tuberculosis. Avg/Pro = average number of matches per protein.

LALIGN E = 1.0	PNEUM	CRM 197	RUB-ELLA	MEAS-LES	MUMPS	Acell PERT	DIPH	TET	POLIO	Men-ingitis
P0DTC1 Repl 1a	26	4	18	9	6	2	3	1	3	3
P0DTC2 Spike Prot	4	0	5	2	2	0	0	6	1	2
P0DTC3 Prot 3a	2	0	6	1	2	0	0	1	1	0
P0DTC4 Env Prot	0	0	1	0	0	0	0	0	0	0
P0DTC5 Memb Prot	7	2	0	0	1	2	2	1	1	0
P0DTC6 NS6 Prot	0	1	1	0	0	0	0	0	0	0
PODTC7 Prot 7a	0	0	0	0	0	0	0	0	0	0
P0DTC8 NS8 Prot	2	0	0	0	0	0	0	0	0	0
P0DTC9 Nucleoprot	4	1	0	0	1	0	0	0	2	0
P0DTD1 Repl 1ab	6	2	3	0	0	2	0	0	0	0
P0DTD2 NS9b Prot	0	0	0	0	0	0	0	0	0	0
P0DTD3 Prot NS14	0	0	0	0	0	0	0	0	0	0
P0DTD8 Prot 7b	0	0	0	0	0	0	0	0	0	0
Total Matches	51	10	34	12	12	6	5	9	8	5
# Proteins	4	1	6	5	5	9	1	1	7	1
Avg/Prot	12.8	10.0	5.7	2.4	2.4	0.7	5.0	9.0	1.1	5

**Table 4:** Summary of BLAST search result matches between SARS-CoV-2 proteins (left-hand column) and whole bacteria at E = 1.0 (shaded left-hand columns) and E = 10.0 (unshaded right-hand columns): *Bordetella pertussis* (whole PERT); *Mycobacterium tuberculosis* (BCG); *Clostridium leptum* (C. lept); *Escherichia coli* (*E. coli*); *Lactococcus lactis* (L. lact); *Lactobacillus paracasei* (L. para). Note that in contrast to the LALIGN searches (Table 2 and Table 3), the BLAST searches were set to E =1 or E = 10 because of the much larger size of the entire genome as compared with the average of 17 proteins searched for the other vaccines (compare sequences in Figure 3 and Figure 4 to Figure 1 and Figure 2). Avg/Pro = average number of matches per protein.

BLAST, E = 1.0 and 10.0	Whole PERT	BCG	C. lept	*E. coli*	L. lact	L. para	Whole PERT	BCG	C. lept	*E. coli*	L. lact	L. para
P0DTC1 Repl 1a	0	5	0	2	0	1	5	4	4	6	4	3
P0DTC2 Spike Protein	1	0	0	0	0	1	9	4	1	6	1	4
P0DTC3 Protein 3a	0	0	0	1	2	0	10	6	7	5	4	4
P0DTC4 Env Protein	0	0	0	0	0	0	2	0	2	1	0	0
P0DTC5 Memb Prot	1	0	1	1	1	1	2	6	4	6	9	10
P0DTC6 NS6 Protein	1	0	0	0	1	0	4	1	2	0	10	5
PODTC7 Protein 7a	0	0	1	1	0	1	3	2	2	5	6	3
P0DTC8 NS8 Protein	0	0	0	1	0	0	2	1	2	3	1	3
P0DTC9 Nucleoprot	1	0	0	0	0	0	7	4	1	3	2	2
P0DTD1 Repl 1ab	1	0	0	0	1	0	5	4	3	3	4	5
P0DTD2 NS9b	0	0	0	0	0	0	0	3	4	5	3	3
P0DTD3 NS Protein 14	0	0	0	0	0	0	0	1	1	3	0	2
P0DTD8 Protein 7b	1	0	0	0	0	0	6	0	0	0	0	0
Total Matches	6	5	2	6	5	4	55	36	33	46	44	42
# Proteins	3260	3997	2482	4403	2225	2708	3260	3997	2482	4403	2225	2708
Avg/Prot	0.002	0.001	0.001	0.001	0.002	0.002	0.017	0.009	0.013	0.010	0.020	0.016
